# Therapeutic plasma exchange in multiple sclerosis patients with an aggressive relapse: an observational analysis in a high-volume center

**DOI:** 10.1038/s41598-022-23356-w

**Published:** 2022-11-01

**Authors:** R. Bunganic, S. Blahutova, K. Revendova, O. Zapletalova, P. Hradilek, R. Hrdlickova, A. Ganesh, Z. Cermakova, M. Bar, O. Volny

**Affiliations:** 1 Department of Neurology, University Hospital Ostrava, Ostrava, Czech Republic; 2 Department of Clinical Neurosciences, Faculty of Medicine, University Ostrava, Ostrava, Czech Republic; 3 Blood Centre, University Hospital Ostrava, Ostrava, Czech Republic; 4 Department of Clinical Neurosciences, Calgary Stroke Program, University of Calgary, Calgary, Canada

**Keywords:** Neuroscience, Medical research, Neurology

## Abstract

An evidence-based treatment for a Multiple Sclerosis (MS) relapse is an intravenous administration of 3–5 g of Methylprednisolone. In case of insufficient effect or corticosteroids intolerance, the therapeutic plasma exchange (TPE) is indicated. To assess the clinical effect of TPE in treatment of relapse in patients with relapsing-remitting MS (RRMS), we enrolled 155 patients meeting the following criteria (study period: January 2011 to February 2021): (1) age > 18, (2) RRMS according to the McDonald´s 2017 criteria, (3) MS relapse and insufficient effect of corticosteroids/corticosteroids intolerance, (4) baseline EDSS < 8. Exclusion criteria: (1) progressive form of disease, (2) history of previous TPE. Following parameters were monitored: EDSS changes (before and after corticosteroid treatment, before and after TPE; EDSS after TPE was assessed at the next clinical follow-up at the MS Center), and improvement of EDSS according to the number of procedures and baseline severity of relapse. 115 females (74%) and 40 males (26%) were included. The median age was 41 years (IQR 33–47)—131 patients underwent the pulse corticosteroids treatment and TPE, while 24 patients underwent only TPE without any previous corticosteroid treatment. Median baseline EDSS was 4.5 (IQR 3.5–5.5), median EDSS after finishing steroids was 4.5 (IQR 4.0–5.5). EDSS prior to the TPE was 4.5 (IQR 4–6), EDSS after TPE was 4.5 (IQR 3.5–5.5). We observed a significant improvement in the EDSS after TPE (p < 0.001). Sex differences were seen in TPE effectiveness, with median improvement of EDSS in females being −0.5 (IQR 1–0) and in males being 0 (IQR −0.5 to 0), p = 0.048. There was no difference in EDSS improvement by age category: 18–30 years, 31–40 years, 41–50 years, > 50 (p = 0.94), nor by total TPE count (p = 0.91). In this retrospective study of patients with an aggressive relapse and insufficient effect of intravenous corticosteroid treatment, a significant effect of TPE on EDSS improvement was observed. There was no significant difference in TPE effectivity according to the number of procedures, age, nor severity of a relapse. In this cohort, TPE was more effective in females.

## Introduction

Multiple sclerosis (MS) is a complex, chronic autoimmune disease of central nervous system (CNS) that results in demyelination, axonal loss and subsequent neurodegeneration^1^. Affecting nearly 3 million people worldwide^2^, MS is the leading cause of non-traumatic disability in young adults^3^. At the time of onset, the MS manifests as either relapsing–remitting MS (RRMS), which affects 80–85% individuals, or primary progressive MS (PPMS). A dominant feature of RRMS is its characteristic course. It manifests with relapsing pattern, followed by periods of remission, when patients are clinically stable. This course is not only a diagnostic criterion, but it also carries an important prognostic value^4^. Acute inflammation in focal lesions may induce axonal injury, which may lead to irreversible brain and spinal cord atrophy^5,6^. Patients who experienced a relapse may not always reach a complete remission. As a matter of fact, a considerable number of relapses is followed by an incomplete recovery, with 42–49% of relapses resulting in a residual increase in patient’s Expanded Disability Status Scale (EDSS) by at least 0.5 points and 28–33% of relapses resulting in an increase by at least 1 point in EDSS^7,8^.

To treat a relapse, current guidelines recommend an intravenous administration of 500–1000 mg of methylprednisolone for 3–5 days as the first-line therapy, followed by an additional 2000 mg in case of clinical non-responsiveness within 2 weeks after the first corticosteroid treatment. If symptoms persist, the relapse is defined as steroid-resistant and an escalation to therapeutic plasma exchange (TPE) is recommended^9,10^. TPE represents a non-pharmacological treatment that removes a large volume of plasma after separating from the cellular components. After TPE plasma discarded and replaced with a replacement fluid, being either the fresh frozen plasma or 5% albumin^11^. TPE is recommended by the American Academy of Neurology (AAN) as an adjunctive treatment (level B) and by the American Society for Apheresis (ASFA) as Grade 1B (strong recommendation, high-quality evidence)^12,13^. Although the exact mechanism of action in MS remains unclear, patients undergoing relapse may benefit from the direct removal of autoantibodies, immune complexes and cytokines^14^. The very first study focused on the effect of TPE in MS patients was conducted by Khatri et al. in 1985. The results showed higher improvement rate in patients who underwent TPE rather than in a “sham” TPE^15^. Nowadays, a variety of retrospective studies demonstrate improvement rates between 59–87.5%^16^. It is of note, that MS lesions with an immunohistopathological type II pattern predict the best response to TPE^17^. Guidelines of professional societies lack a detailed information on how to perform TPE, e.g. the ASFA recommends a total of 5–7 procedures over a period of 10–14 days with exchange of 1–1.5-fold plasma volume^12^.

The data about TPE efficacy is not consistent, randomized controlled trials investigating the adjunct effect of TPE in steroid-resistant MS relapse are lacking and official guidelines do not provide further information on how to perform TPE, e.g. how to proceed according to the severity of relapse^18,19^.

The main objective of this study is to evaluate an adjuvant effect of TPE in management of severe and/or steroid-resistant acute MS relapse.

## Methods

All methods were carried out in accordance with relevant guidelines and regulations, including The Strengthening the reporting of Observational Studies in Epidemiology (STROBE) guidelines for reporting observational studies. This study was approved by the Ethical Committee of University Hospital Ostrava under the reference number 519/2021 (informed consent is waived by this committee). For this single center retrospective study, 155 patients with RRMS who received their first TPE treatment between 2011 and 2021 at the University Hospital Ostrava (Czech Republic) were enrolled. Inclusion criteria were: a diagnosis of RRMS according to the 2017 revised McDonald´s criteria, age > 18 years, first acute relapse with a lack of or incomplete improvement after the intravenous methylprednisolone (IVMP) pulse therapy or a history of corticosteroid intolerance. An aggressive relapse was defined as a relapse with an increase in EDSS by 2.5 points or more, or an increase of 2 degrees in 3 or more in functional subscales (FS), or 3 degrees in 1 FS. It can also be defined by the necessity of hospitalization, if this hospitalization is not explained by another complicating disease or other reason than the severity of the relapse^20^.Patients with a history of previous TPE and/or progressive form of MS were not included. Additionally, patients with insufficient or missing data were excluded. Monitored clinical parameters were: time to TPE initiation, EDSS dynamics (before and after corticosteroid treatment, before and after TPE; EDSS after TPE was assessed at the next clinical follow-up at the MS Center), and improvement of EDSS according to the number of procedures and severity of relapse. The EDSS after TPE was evaluated by MS specialists at our high-volume MS center. For further analysis, patients were divided into subgroups according to their gender, age, baseline EDSS and total number of TPE procedures.

TPEs were performed according to the ASFA and European society for hemapheresis (ESFH) guidelines via peripheral venous access (PVA), achieved via either two large peripheral veins or a dual-lumen central-line catheter (CVC), in a high-volume Apheretic Center at the University hospital Ostrava, where over 1000 TPEs are performed annually. In our hospital, an attending neurologist indicates the TPE, while a specialized team of Apheretic center performs the TPE procedure. Usually, a series of 3 to 5 TPEs each carried out every other day is performed. The centrifugal separators COM.TEC (Fresenius Kabi) and Spectra Optia (Terumo BCT) with continual separation program are used with the blood flow set between 50 and 70 mL/min. There is no need for specific premedication before the TPE in patients with RRMS. To prevent hypotension, it is recommended to omit a dose of anti-hypertension medication 24 h prior to TPE. Anticoagulation treatment before TPE is not considered a contraindication for TPE procedure.

The separator is normally set for a change of one plasma volume (total plasma volume/TPV). The volume of collected plasma is calculated automatically according to the patient height, weight, gender and actual hematocrit. The combination of 1/3 of normal saline and 2/3 of 5% human albumin is used as a replacement fluid. The balance rate is set on 100–115% (median 110%). We use higher volume of replacement fluids compared to collected plasma volume to prevent a periprocedural as well as postprocedural hypotension. The anticoagulant citrate dextrose solution USP formula A (ACD-A) is used as an anticoagulant with the anticoagulant-to-whole blood ratio 1:12. An intravenous calcium gluconate (10 mL/h) is used to avoid hypocalcemia during the whole separation process.

### Statistical analysis

The study data were analyzed using the Stata software version 14 (StataCorp LLC). The quantitative data are reported as medians and interquartile range (IQR), means and standard deviations (SD). The qualitative data were evaluated using the frequency tables. For calculating the difference between EDSS before and after TPE, the Wilcoxon test was used. The Kruskal–Wallis equality-of-populations rank test was used to assess the differences of EDSS improvement among the predefined subgroup (baseline EDSS, total number of procedures and age category). The TPE efficacy according to gender was tested using the Mann–Whitney test. To assess how the EDSS changed over time from initial presentation, past the steroid course (for those treated with steroids), and from before to after the TPE course, we used repeated-measures analyses with mixed-effects linear regression with the main covariates being age, sex, receipt of methylprednisolone, and baseline EDSS. In addition, we evaluated how the EDSS trajectory was modified by age, sex, and receipt of methylprednisolone by repeating the mixed-effects linear regression with an interaction term for the timing of EDSS assessment and the variable of interest (age grouped into quartiles, sex-female vs male, methylprednisolone-yes vs no). The α–level was set to α = 0.05, for a corresponding P–value smaller than α (P < 0.05), the results were considered as a statistically significant.

All methods were carried out in accordance with The Strengthening the Reporting of Observational Studies in Epidemiology (STROBE) guidelines for reporting observational studies.

## Results

**Figure 1:**
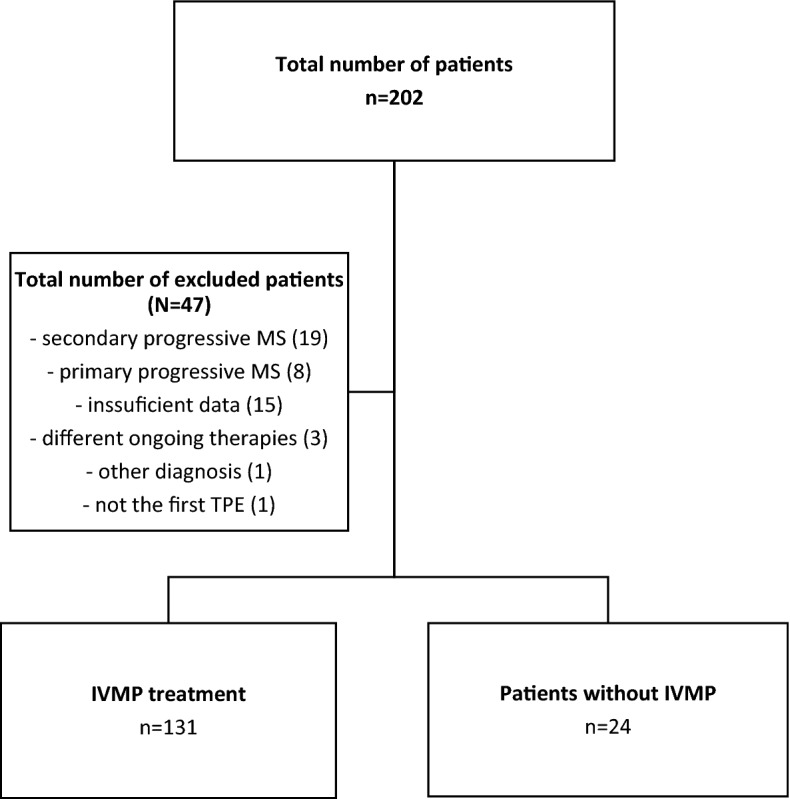
Study flowchart. Legend: MS—multiple sclerosis, TPE—therapeutic plasma exchange, IVMP—methylprednisolone intravenous treatment.

We retrospectively analyzed a total number of 603 TPEs in 155 RRMS patients. TPEs were performed between January 2011 and February 2021. The median age was 41 years (IQR 33–47). Six hundred and one TPEs (98%) were performed via the PIVC, while only 3 patients (2%) had a central venous catheter inserted due to a PVA failure. Three hundred and eighty-one (63%) TPEs were performed in out-patient (ambulatory) setting, while 222 (37%) as in-patient. One hundred and thirty-one patients (85%) had a previous methylprednisolone treatment, while 24 (15%) patients with a history of corticosteroid intolerance received TPE only. Study flowchart is presented in Fig. 1 and analyzed sub/groups are presented in Table 1.

**Table 1:** Analyzed groups and subgroups.

Group	Subgroups	Number	p
Sex	Male	40 (26%)	0.0488 (A)
	Female	115 (74%)	
Age	18–30	24 (15%)	0.9399 (B)
	31–40	51 (33%)	
	41–50	54 (35%)	
	51 +	26 (17%)	
Baseline EDSS	0–3	28 (18%)	0.1458 (B)
	3.5–6	103 (67%)	
	6 +	24 (15%)	
Number of procedures	3	77 (50%)	0.9053 (B)
	4	29 (19%)	
	5	39 (25%)	
	6 +	10 (6%)	

A—Two-sample Wilcoxon rank–sum test, B—Kruskal–Wallis equality of populations rank test.

The separators were set for an exchange of 1.0 TPV, median blood volume processed was 5067 ml (IQR 4318–6355). Median applied dose of ACD-A was 415 ml (IQR 354–520). Median separation time was 83 min (IQR 73–100). TPE parameters are presented in Table 2.

**Table 2:** TPE parameters.

	Median (IQR)
Total blood volume (TBV), ml	4620 (4095–5553)
Total plasma volume (TPV), ml	2885 (2541–3323)
Balance rate	110 (110–110)
Separation time, min	83 (73–100)
ACD-A volume, ml	415 (354–520)
Volume of substituted 5% human albumin per TPE, ml	1750 (1500–2000)

ACD-A—Anticoagulant Citrate Dextrose Solution A.

**Figure 2:**
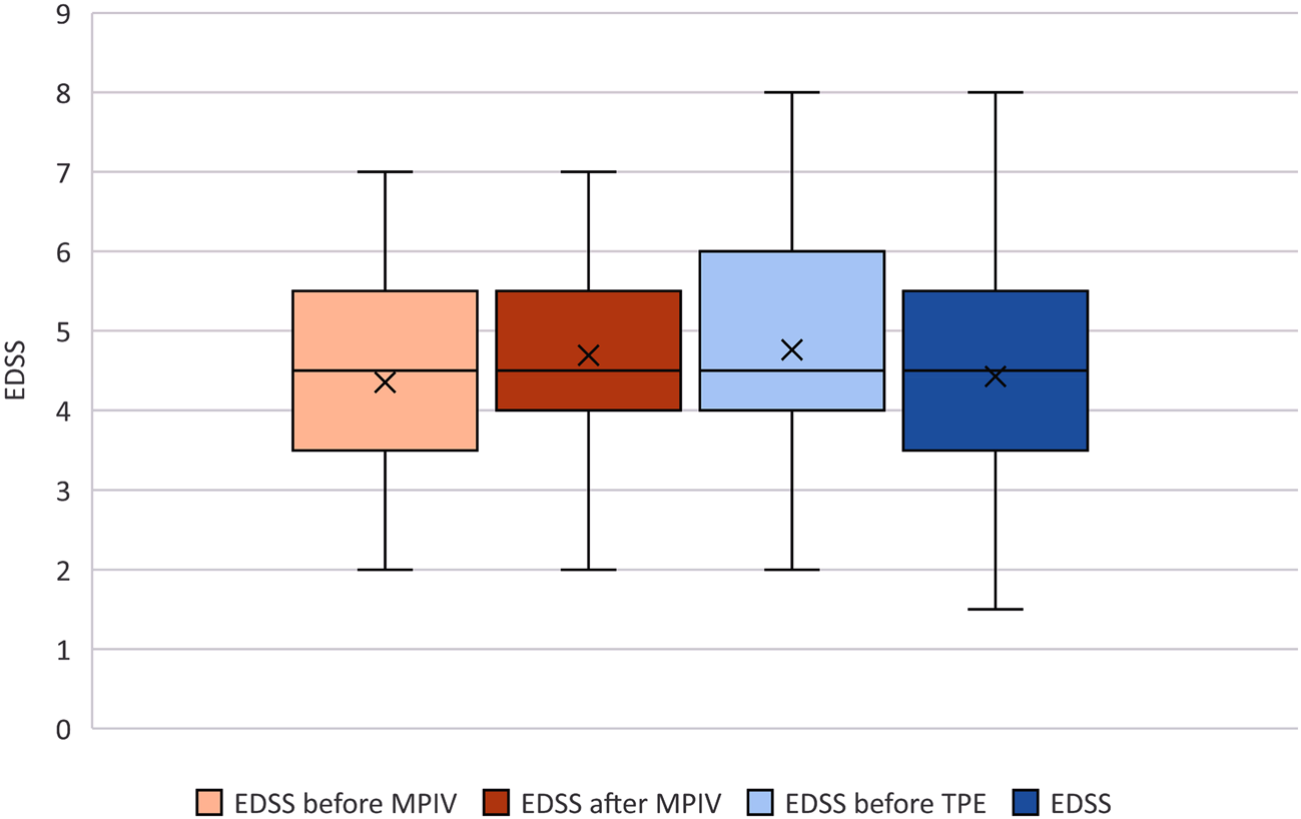
Changes in EDSS scores over the course of the study. Legend: Time 0–before corticosteroids, Time 1–after corticosteroids, Time 2–before TPE, Time 3–after TPE. The graph includes everyone (n = 155), but only patients who got corticosteroids contribute to times 0 and 1 (n = 131).

Out of all 155 patients treated with TPE, 77 of them (50%) experienced an improvement in their EDSS. EDSS of 58 patients (37%) remained unchanged compared to their baseline, while 20 patients (13%) experienced further worsening of their clinical status. Median baseline EDSS was 4.5 (IQR 3.5–5.5), median EDSS after IVMP treatment was 4.5 (IQR 4–5.5), median EDSS prior to the TPE was 4.5 (IQR 4–6). To assess how the EDSS changed over time from initial presentation, past the steroid course (for those treated with steroids), and from before to after the TPE course, we used the repeated-measures analyses with mixed-effects linear regression with the main covariates being age, sex, receipt of methylprednisolone, and baseline EDSS. Using this model, we proved that the changes in EDSS dynamics were significant (p < 0.001 for general trend over time). Figure 2 presents the dynamics of EDSS. Median time until the next clinical visit was 6.6 weeks (IQR 3.8–11.2). Median EDSS after TPE was 4.5 (IQR 3.5–5.5). Median EDSS difference, calculated as EDSS after TPE minus baseline EDSS, was 0 (IQR –1–0). Median time to TPE initiation was 49 days (IQR 19 – 79).

We observed a significant difference between the baseline and post-TPE EDSS (p < 0.001). Different effectivity of TPE according to sex was found, with median improvement of EDSS in females being –0.5 (IQR –1–0) and in males being 0 (IQR –0.5–0), p = 0.048. There was no difference in EDSS improvement according to the age category: 18–30 years, 31–40 years, 41–50 years, > 50 (p = 0.94), or by a total number of TPE (p = 0.91).

### TPE side effects/adverse events

The majority of treated patients (94%) were free of any adverse events (AE). The most common AE was a PVA failure, which occurred in 22 patients; 19 patients needed a reinsertion of PVA, while a central venous catheter was needed in 3 patients (0.5%). Vasovagal reactions occurred in 1.8% patients. Allergic reactions were observed and treated accordingly in 3 patients (0.5%). Technical problems with set occurred in 1 case (0.2%).

## Discussion

In this retrospective study focused on the effect of TPE on EDSS improvement in patients with a steroid-resistant relapse of RRMS, we observed that 50% patients reached a significant EDSS improvement, 37% of patients remained unchanged in their EDSS, while 13% patients experienced further clinical worsening. Females responded better to the TPE than males in our cohort. We did not observe a significant difference in TPE effectivity according to the severity of baseline EDSS or a total number of TPE procedures.

The ASFA recommends TPE for a treatment of aggressive MS relapse to the category II, meaning that it is accepted as a second-line therapy. The AAN recommends TPE as an adjunctive therapy (level B)^12,13^. A recent systematic review and meta-analysis^16^ focused on TPE in RRMS systematically analyzed 14 studies (mostly retrospective) of a different methodological quality and heterogeneity of patient cohorts. These studies differed also in the numbers of patients, TPE protocols (some of them even do not mention the TPE protocols), number of TPE procedures and TPV. Our study focused solely on the patients with RRMS.

Our study analyzed 603 TPEs performed in 155 patients, which represents one of the largest cohorts so far. In all 155 patients, it was their first TPE treatment. The above-mentioned met-analysis included 398 patients from 14 studies. The six studies were considered to be methodologically similar to our study^3,18,21–24^. Nevertheless, most of the primary studies selected the target neurological deficit (TND) as a primary endpoint^21–23^, one study used the MSFC^18^ and one study used the EDSS as an outcome measure^24^. The EDSS was considered as a secondary outcome in most of the studies^18,21,23^. In our study, we used the EDSS as a primary outcome, as it still represents the most commonly used clinical scale in MS patients. Since it was evaluated by certified MS specialists on every follow-up, it allowed us to monitor the EDSS dynamics over time (before corticosteroid treatment, after corticosteroid treatment, before initiation of TPE and after the TPE treatment/at the next clinical follow-up). Even though the TND and MSFC are considered being more sensitive scales to detect changes in patient clinical status, unfortunately we lacked sufficient data to evaluate these outcomes accordingly.

The data from the meta-analysis suggest effectivity of TPE between 41–93%, mostly around 76%^16^. In our cohort, we observed therapy response in 50% patients. The relatively lower percentage of improvement might be explained by selecting EDSS as less sensitive scale for addressing relapse. Considering the number of patients included, methodological background, number of TPEs and major findings, the study by *Ehler *et al*.*^23^ was similar to our study. They included MS patients with a history of steroid-intolerance and/or inadequate response to steroids. The study differed in the primary endpoint (TND). Significant therapy response was observed in 72% patients, which is higher than in 34% patients who experienced EDSS improvement in our cohort.

Considering the safety of TPE, even though many adverse events have been described, they are usually preventable and thus rare in occurrence. What we cannot predict is an allergic reaction or technical problems with the TPE set. The occurrence of adverse events in literature ranges between 4 to 26%^16^. In our cohort, only 6% of adverse events were reported, most of them accounting for a PIVC failure (3.7%), followed by a vasovagal reaction (1.8%) and allergic reactions (0.5%), and all of them were not considered as severe.

Though commonly used, TPE is not the only way to treat a steroid-refractory MS relapse. The first successful use of immunoadsorption (IA) in MS relapse was described in 2000 by *De Andrés et* *al.*^25^. Since then, several studies have compared IA to TPE as a new, possibly safer and more effective alternative of a second-line therapy in MS. MS patients, who underwent IA, showed a higher response rate after 4 weeks (86.7%) compared to patients treated with the TPE (76.7%). A study by *Lipphardt *et al*.* conducted in 2018 did not support these findings and claimed, that IA and TPE seemed to be equally safe and effective in steroid-resistant MS relapse. Further well-designed studies are needed. The problem that arises with IA is that in many countries the costs are not covered from the health insurance and thus it limits the use of IA.

Single center and retrospective design of our study represent major limitations. Unmeasured confounders may have affected treatment decisions. Additionally, the EDSS assessment was not blinded, as it was performed by different certified MS specialists (who were not aware about this project at the time of clinical assessment). The incompleteness of hospital records (including the change of hospital information system within the study period) and data collection from retrospective chart review limit further analyses, comparisons and conclusions (e.g. incomplete or missing data on EDSS subscales). Another limitation was that there was no separate control group. Since well-designed randomized control trials are lacking and most of the published studies suffer from several limitations, treating severe MS relapse still remains a challenge^3,26^.

## Data Availability

Anonymized data will be shared by reasonable request from a qualified investigator from either the first and/or corresponding authors.
